# Molecular profiling of angiogenesis in hypericin mediated photodynamic therapy

**DOI:** 10.1186/1476-4598-7-56

**Published:** 2008-06-13

**Authors:** Ramaswamy Bhuvaneswari, Yik Y Gan, Sasidharan S Lucky, William WL Chin, Seyed M Ali, Khee C Soo, Malini Olivo

**Affiliations:** 1 Division of Medical Sciences, National Cancer Centre Singapore, 11 Hospital Drive, 169610, Singapore; 2 Natural Sciences and Science Education, National Institute of Education, Nanyang Technological University, 1 Nanyang Walk, 637616, Singapore; 3 Singapore Bioimaging Consortium, Biomedical Sciences Institutes, 11 Biopolis Way, #02-02 Helios, 138667, Singapore

## Abstract

**Background:**

Photodynamic therapy (PDT) involves the administration of a tumor-localizing photosensitizing drug, which is activated by light of specific wavelength in the presence of molecular oxygen thus generating reactive oxygen species that is toxic to the tumor cells. PDT selectively destroys photosensitized tissue leading to various cellular and molecular responses. The present study was designed to examine the angiogenic responses at short (0.5 h) and long (6 h) drug light interval (DLI) hypericin-PDT (HY-PDT) treatment at 24 h and 30 days post treatment in a human bladder carcinoma xenograft model. As short DLI targets tumor vasculature and longer DLI induces greater cellular damage, we hypothesized a differential effect of these treatments on the expression of angiogenic factors.

**Results:**

Immunohistochemistry (IHC) results showed minimal CD31 stained endothelium at 24 h post short DLI PDT indicating extensive vascular damage. Angiogenic proteins such as vascular endothelial growth factor (VEGF), tumor necrosis growth factor-α (TNF-α), interferon-α (IFN-α) and basic fibroblast growth factor (bFGF) were expressed to a greater extent in cellular targeting long DLI PDT compared to vascular mediated short DLI PDT. Gene expression profiling for angiogenesis pathway demonstrated downregulation of adhesion molecules – cadherin 5, collagen alpha 1 and 3 at 24 h post treatment. Hepatocyte growth factor (HGF) and Ephrin-A3 (EFNA3) were upregulated in all treatment groups suggesting a possible activation of c-Met and Ephrin-Eph signaling pathways.

**Conclusion:**

In conclusion, long DLI HY-PDT induces upregulation of angiogenic proteins. Differential expression of genes involved in the angiogenesis pathway was observed in the various groups treated with HY-PDT.

## Background

Photodynamic therapy (PDT) is a cytotoxic treatment, predominantly used in anti-cancer approaches, that depends on the retention of photosensitizers in tumor and its subsequent activation by light [1]. The tumor response to PDT is complex; involving vascular damage, direct tumor cell death, and the induction of immune responses that greatly depend on the photosensitizer pharmacokinetics and the treatment conditions [2]. Bladder cancer is the fifth most common malignancy in Europe and the fourth most in the United States, and the main thrust of research is to prevent the progression of superficial cancers to metastatic tumors [3]. PDT has been shown to induce complete or partial destruction of tumor, reducing recurrence rate in bladder cancer patients [4]. Hypericin (HY), a perylenequinone with potent photosensitizing properties induces *in vivo *and *in vitro *photocytotoxic activity and displays potential as a photosensitizer for PDT [5-7]. Our previous study reported the use of HY as a selective marker for the diagnosis of bladder tumors in clinical trials [8].

The preference of vascular versus cellular targeting of PDT treatment is highly dependent upon the relative distribution of the photosensitizer in the vascular and cellular compartments and can also be effectively manipulated by varying the drug and light intervals (DLI). Initially after photosensitizer is administered, the drug is confined within the tumor vasculature and employing a short DLI largely damages the tumor vasculature. However during longer DLI the photosensitizer diffuses out from the blood vessels into the tissue, and accumulates in the tumor cellular compartment and the subsequent light irradiation targets the cells and causes tumor cytotoxicity [9-11]. Vascular damage has been implicated as the primary antitumoral effect in PDT with various photosensitizers [12], including HY [10]. An earlier study by our group had shown that vascular damage and direct cell killing together with a strong inflammatory response contribute towards tumor necrosis and shrinkage following PDT in nasopharyngeal tumors [13]. It is believed that the circulating photosensitizer in the plasma generates cytotoxic reactive oxygen species, which leads to primary vascular damage that result in tumor necrosis. It has also been acknowledged that targeting tumor vasculature proves to be a promising approach in cancer treatment [14].

As part of the PDT process, hypoxia is induced in tumors and this oxidative stress initiates a variety of molecular and physiological responses that could potentially lead to neovascularisation. Angiogenesis, the formation of new blood vessels from existing vasculature, is a multistep process involving the degradation of extracellular matrix, endothelial cell proliferation, migration and tube formation [15,16]. Our studies as well as other reports have observed the overexpression of angiogenic markers after PDT treatment [17-20]. The response of both vascular and cellular targeted PDT and the combination of both has been well documented by Chen et al. [9-11]. Though previous studies have shown different mechanistic aspects of vascular and cellular mediated PDT, little is known about the angiogenic responses in these treatment variations. In this study we investigated the effect of short and long DLI in modulating the mechanism of angiogenesis in tumors. Our results revealed differential expression of several proteins and genes involved in various angiogenic processes, i.e. proliferation, inflammation and carcinogenesis in response to HY-PDT of bladder carcinoma.

## Results

### Hypericin macrofluorescence

**Figure 1:**
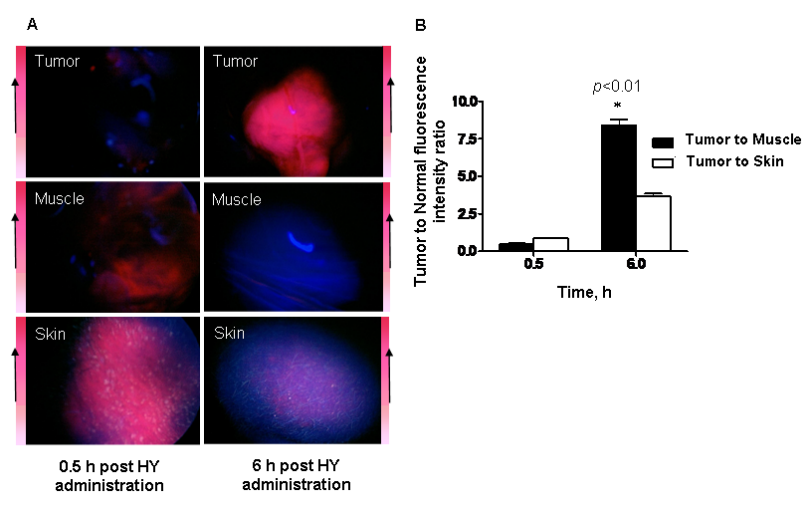
**A**, Representative red fluorescence images of tumor, muscle and skin at 0.5 h and 6 h post HY administration shows increased uptake in the tumor at 6 h time point compared to 0.5 h. **B**, Tumor to normal tissue ratio was greater at 6 h compared to 0.5 h post HY administration, *p *< 0.01, indicating greater tumor uptake. Each group represents the mean (bars, SE) of 8 animals.

Hypericin fluorescence in the tumor was evaluated using a fluorescence endoscopy system to determine the uptake kinetics in the tumor at 0.5 and 6 h post drug administration. Fluorescence intensity in the tumor was higher at 6 h compared to 0.5 h post HY administration suggesting higher tumor selectivity (Fig. 1A). At 6 h fluorescence in muscle and skin was lower indicating higher clearance from normal tissue. The tumor to normal tissue ratio tabulated from the fluorescence images was greater at 6 h compared to 0.5 h post HY administration, *p *< 0.01 (Fig. 1B).

### Hypericin microfluorescence

**Figure 2:**
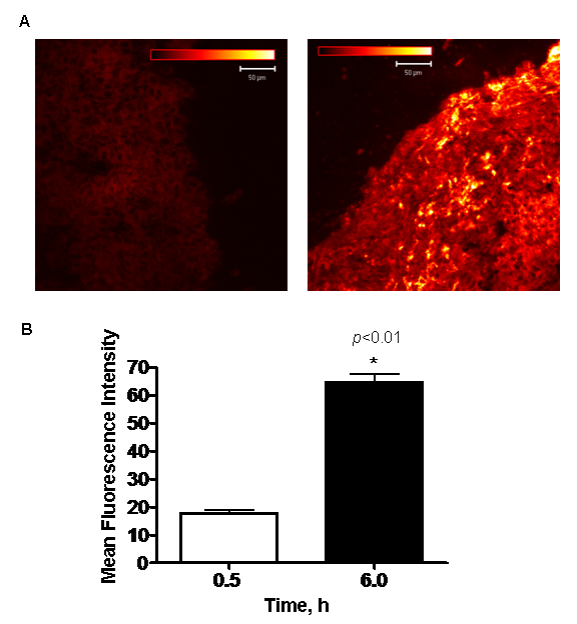
**A**, Microfluorescence of HY in the tumor at 0.5 h and 6 h was detected using confocal laser scanning microscope. HY fluorescence in the tumor was increased at 6 h compared to 0.5 h time point. **B**, In correlation with the macrofluorescence data, quantification of HY fluorescence at 0.5 h and 6 h clearly indicated greater uptake at 6 h, *p *< 0.01. Each group represents the mean (bars, SE) of 8 animals.

Hypericin microfluorescence was detected using a confocal laser scanning microscope to establish the correlation between macro and microfluorescence observed in the tumors at 0.5 and 6 h post drug administration. Microscopic fluorescence of HY in the tumor was significantly higher at 6 h compared to 0.5 h time point (Fig. 2A). Fluorescence increased twofold at 6 h post HY administration compared to 0.5 h, *p *< 0.01 (Fig. 2B).

### Tumor volume assessment

**Figure 3:**
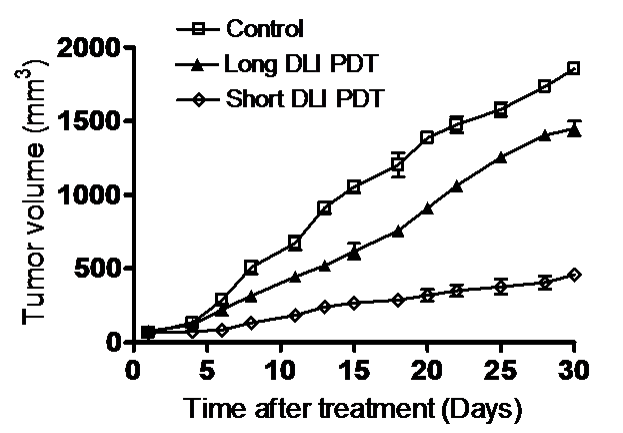
Tumor volume charted against treatment days for control, short DLI and long DLI HY-PDT treated bladder carcinoma xenografts. The tumors treated with short DLI PDT exhibited greater tumor control compared to long DLI PDT (*p *< 0.05) and control (*p *< 0.01). Each group represents the mean (bars, SE) of 10 animals.

Tumors were allowed to grow up to sizes of 6–7 mm in diameter before PDT treatment was carried out, and were measured three times a week. Animals subjected to vascular targeted short DLI PDT exhibited greater tumor response compared to cellular targeted long DLI PDT, *p *< 0.05 and control, *p *< 0.01 (Fig. 3). The decrease in growth rate in short DLI PDT group is indicative of significant damage to the tumor vasculature during treatment.

### Immunohistochemistry

**Figure 4:**
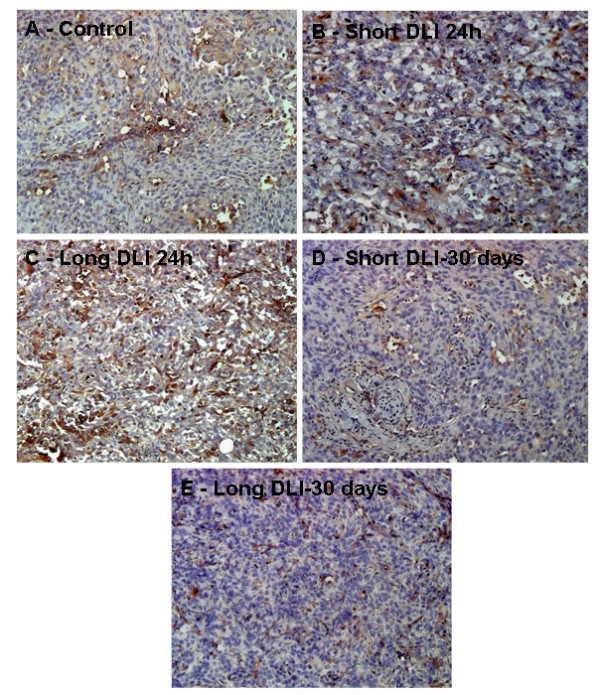
CD31 expression was assessed in tumors treated with various treatment regimens using immunohistochemistry. **A **– Control (untreated tumor), **B **– Short DLI 24 h, **C **– Long DLI 24 h, **D **– Short DLI 30 days, **E **– Long DLI 30 days. Control, short DLI 30 days and long DLI 30 days tumor sections exhibited 8 to 10% staining. In short DLI PDT at 24 h 10% staining of damaged vessels were noted. Around 51% CD31 expression was observed in the long DLI 24 h PDT group, (magnification ×200).

Blood vessel staining was performed to calculate vessel density after HY-PDT treatment. Immunohistochemistry revealed highest endothelium density at 24 h after long DLI PDT (IHC score 4) (Fig. 4C), however at 24 h post short DLI PDT, lesser smudged and diffused staining pattern (IHC score 2) of endothelial cells were noted (Fig. 4B). Moderate staining (IHC score 1) of blood vessels were observed in control, short and long DLI PDT 30 days treated tumors (Fig 4A, D, E).

### Western immunoblotting

**Figure 5:**
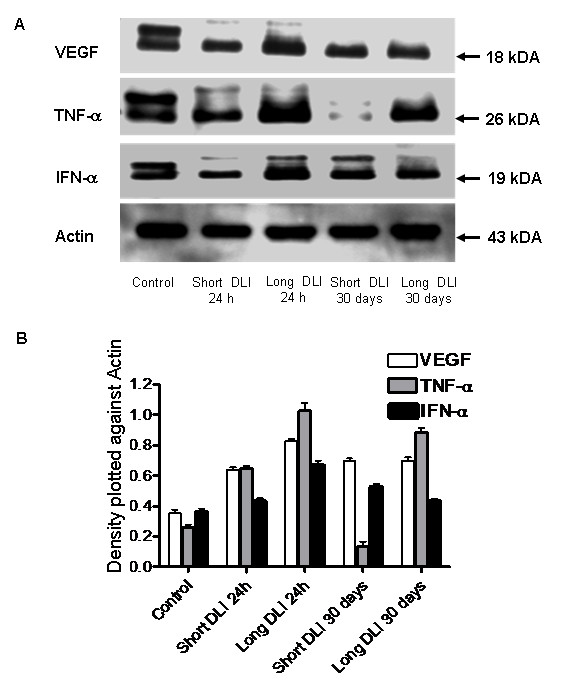
**A **– Expression of VEGF, TNF-α and IFN-α was detected in the tumors using western immunoblot analysis. Expression of actin was used to monitor protein loading. Higher protein expression was detected in cellular targeted long DLI PDT at 24 h compared to short DLI PDT at 24 h. TNF-α is secreted predominantly by the macrophages and its downregulaton at short DLI 30 days could suggest minimal macrophage recruitment due to extensive vascular damage. At 30 days short and long DLI PDT comparable levels of IFN-α and VEGF was noticed. **B **– Ratio of VEGF, IFN-α and TNF-α density was plotted against actin. Compared to control, expression of proteins were statistically significant (*p *< 0.05) for all the groups except IFN-α at long DLI 30 days PDT group. Each group represents the mean (bars, SE) of 8 tumors.

Blotting of angiogenic proteins, i.e. VEGF, TNF-α and IFN-α was carried out to understand the modulations in protein expression at 24 h and 30 days post PDT treatment. TNF-α was significantly downregulated at 30 days post vascular targeted short DLI PDT compared to all other treatment groups. Expression of VEGF and IFN-α was significantly upregulated in long DLI 24 h group compared to all other groups (*p *< 0.05) (Fig 5A, B).

### Detection of angiogenic proteins using antibody arrays

**Figure 6:**
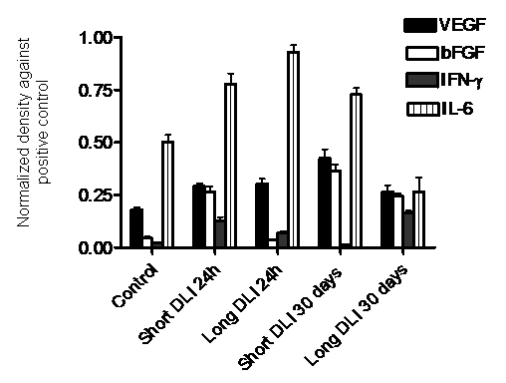
Expression of angiogenic proteins in the treated tumors were analysed using antibody arrays. Density of proteins were plotted and normalised against the positive control Actin. Each group represents the mean (bars, SE) of 8 tumors (i.e. one membrane was used per tumor). Compared to control tumors, VEGF secretion was significantly enhanced in all treatment groups (*p *< 0.05). In a similar way, compared to control tumors, greater bFGF secretion was noted in all groups (*p *< 0.05) except long DLI PDT at 24 h. IFN-γ expression was highest at 30 days post long DLI treatment. The inflammatory protein, IL-6 was upregulated in all the treatment groups except long DLI 30 days PDT.

Expression of major angiogenic proteins i.e., VEGF, bFGF, IFN-γ and IL-6 were detected in the treatment groups using human angiogenesis antibody arrays. VEGF expression was significantly greater (*p *< 0.05) in the treatment groups compared to control tumors. bFGF expression was significantly greater in vascular mediated short DLI PDT compared to cellular mediated long DLI PDT groups. IFN-γ expression was most pronounced at 30 days in the long DLI PDT group. Upregulation of inflammatory protein IL-6 was remarkably higher compared to all other angiogenic proteins. At 24 h post PDT significantly high levels of IL-6 were expressed (Fig. 6).

### Real-Time PCR to detect expression of genes involved in angiogenesis pathways

Gene expression of different genes involved in angiogenesis was assessed to understand the role of transcriptional factors before tumor regrowth. Adhesion molecules i.e., cadherin 5 (CDH5) and collagens (COL18A1 and COL4A3) were downregulated 24 h post vascular targeted short DLI PDT and cellular targeted long DLI PDT, however at 30 days post treatment CDH5 and COL18A1 were upregulated with the exception of COL4A3. Intergrin alpha V (ITGAV) and Intergrin beta 3 (ITGB3) were downregulated in short and long DLI PDT at 24 h, however increased mRNA transcription was noticed at 30 days time point with the exception of ITGAV that was downregulated at short DLI PDT. Ephrin A3 (EFNA3) and Ephrin B2 (EFNB2) are ligands of the eph receptor family that plays a major role in tumor remodeling. Ephrin A3 was upregulated at 24 h post PDT, however ephrin B2 was downregulated. Both EFNA3 and EFNB2 were overexpressed 30 days post PDT treatment. Hepatocyte growth factor (HGF) expression was found to be 4-fold greater than epidermal growth factor (EGF) at 24 h post vascular mediated short DLI PDT. HGF acts through the Met-receptor pathway to promote angiogenesis and its transcription was well pronounced in all our PDT treated groups. EGF induced signaling has often been associated with tumor invasion and metastasis. In our experiments, EGF was significantly overexpressed at 24 h in vascular targeting short DLI PDT and was downregulated in all other treatment groups. Increased expression of platelet-derived growth factor alpha (PDGFA) was observed in all the treatment groups. Neuropilin 2 (NRP2), a nonsignaling transmembrane receptor was suppressed only at 30 days post long DLI PDT. The notch ligand Jagged 1 (JAG1) was downregulated in all the treatment groups. TEK tyrosine kinase (TEK) was significantly upregulated at 30 days post short DLI PDT only. Angiogenesis growth factors such as transforming growth factor alpha (TGFA) and vascular endothelial growth factor C (VEGF-C) were downregulated 24 h post PDT but were upregulated by 30 days. Tumor necrosis factor, alpha induced protein 2 (TNFAIP2) was downregulated only at 24 h post long DLI PDT. The function of each gene is provided in Table 1, and fold change and *p*-values for the genes are provided in Tables 2 and 3.

**Table 1:** Gene expression profiling was performed using Human Angiogenesis real time PCR Array.

**Gene Description**	**Unigene**	**Gene Bank**	**Function**
Cadherin 5, type 2, VE-cadherin	Hs.76206	NM_001795	Plays an important role in cell adhesion and is crucial for vessel growth and maturation.
Collagen, type XVIII, alpha 1	Hs.517356	NM_030582	Component of extra-cellular matrix (ECM) that is important for tissue organization.
Collagen, type IV, alpha 3	Hs.570065	NM_000091	
Integrin, alpha V	Hs.436873	NM_002210	Cell adhesion molecules that plays a key role in tumor growth, angiogenesis and metastasis.
Integrin, beta 3	Hs.218040	NM_000212	
Hepatocyte growth factor	Hs.396530	NM_000601	Stimulates tumor cell motility, invasion and angiogenesis and its receptor is a tyrosine kinase encoded by c-met.
Ephrin-A3	Hs.516656	NM_004952	Ephrins are cell-membrane-bound ligands for Eph receptor tyrosine kinases and regulate a variety of developmental processes.
Platelet-derived growth factor alpha polypeptide	Hs.645488	NM_002607	Stimulates endothelial cell proliferation.
Epidermal growth factor	Hs.419815	NM_001963	An EGFR ligand, regulates cell growth, proliferation and differentiation.
Neuropilin 2	Hs.471200	NM_003872	Receptor for VEGF and semaphorin (SEMA) and is involved in angiogenesis and neuronal guidance.
Ephrin-B2	Hs.149239	NM_004093	An Eph ligand, promotes cell attachment, cell-adhesion, migration, capillary tube formation and tumor angiogenesis.
Jagged 1	Hs.224012	NM_000214	A notch ligand, it regulates a transmembrane ligand-receptor signaling pathway involved in the regulation of endothelial cell differentiation.
Tumor necrosis factor, alpha-induced protein 2	Hs.525607	NM_006291	A cytokine mainly produced by macrophages causes apoptotic cell death, cellular proliferation, differentiation and inflammation.
TEK tyrosine kinase, endothelial	Hs.89640	NM_000459	Helps in vascular maturation, maintenance of integrity and remodeling.
Transforming growth factor, alpha	Hs.170009	NM_003236	Binds to EGFR and is involved in tumorigenesis.
Vascular endothelial growth factor C	Hs.435215	NM_005429	Involved in angiogenesis and lymphangiogenesis.

Selected genes that are involved in the angiogenesis pathway in HY-PDT treated tumors are listed below. The gene description, unigene entry, gene bank code and the function of each gene are described in the table.

**Table 2:** Gene expression profiling was performed for HY-PDT treated tumors.

**Gene**	**Gene Symbol**	**Short DLI PDT 24 h**			**Long DLI PDT 24 h**		
			
		**Fold change**	**p-value**	**Up(↑)/down (↓) regulation**	**Fold change**	**p-value**	**Up (↑)/down (↓) regulation**
***Adhesion molecules***							

Cadherin 5, type 2, VE-cadherin	CDH5	0.080	0.015	↓	0.214	0.0313	↓
Collagen, type XVIII, alpha 1	COL18A1	0.016	0.0335	↓	0.024	0.051	↓
Collagen, type IV, alpha 3	COL4A3	0.006	0.0075	↓	0.027	0.027	↓
Integrin, alpha V	ITGAV	0.0007	0.00003	↓	0.086	0.029	↓
Integrin, beta 3	ITGB3	0.169	0.027	↓	0.144	0.025	↓

***Growth factors and receptors***							

Hepatocyte growth factor	HGF	30.70	0.00008	↑	8.04	0.0014	↑
Ephrin-A3	EFNA3	12.76	0.026	↑	12.47	0.035	↑
Platelet-derived growth factor alpha polypeptide	PDGFA	10.15	0.038	↑	22.68	0.0109	↑
Epidermal growth factor	EGF	7.013	0.003	↑	0.051	0.017	↓
Neuropilin 2	NRP2	5.502	0.031	↑	5.993	0.012	↑
Ephrin-B2	EFNB2	0.003	0.0002	↓	0.205	0.039	↓
Jagged 1	JAG1	0.001	0.008	↓	0.004	0.021	↓
Tumor necrosis factor, alpha-induced protein 2	TNFAIP2	3.34	0.045	↑	0.136	0.031	↓
TEK tyrosine kinase, endothelial	TEK	0.250	0.042	↓	0.218	0.0028	↓
Transforming growth factor, alpha	TGFA	0.054	0.030	↓	0.018	0.0106	↓
Vascular endothelial growth factor C	VEGFC	0.0031	0.003	↓	0.023	0.013	↓

The gene symbol, fold-change, *p*-value and the status of transcription have been described for each gene. The genes involved in the angiogenesis pathway were divided into two groups – adhesion molecules and growth factors and receptors. Six genes regulating the angiogenesis pathway i.e., HGF, EFNA3, EGF, NRP2, PDGFA and TNFAIP2 were upregulated in both short and long DLI 24 h HY-PDT with the exception of EGF in long DLI HY-PDT and 10 genes CDH5, COL18A1, COL4A3, EFNB2, ITGAV, ITGB3, TEK, TGFA, VEGFC and JAG1 were downregulated in both short and long 24 h HY-PDT when compared with the controls (*p *values are given in the table).

**Table 3:** At 30 days post HY-PDT 12 genes COL18A1, CDH5, ITGB3, VEGFC, HGF, TGFA, PDGFA, TEK, EFNA3, EFNB2, NRP2 and TNFAIP2 exhibited higher transcriptional activity at both short and long DLI 30 days PDT with the exception of NRP2 and TEK in cellular mediated long DLI PDT at 30 days.

**Gene**	**Gene Symbol**	**Short DLI PDT 30 days**			**Long DLI PDT 30 days**		
			
		**Fold change**	**p-value**	**Up (↑)/down (↓) regulation**	**Fold change**	**p-value**	**Up (↑)/down (↓) regulation**
***Adhesion molecules***							

Collagen, type XVIII, alpha 1	COL18A1	46.21	0.041	↑	39.40	0.047	↑
Cadherin 5, type 2, VE-cadherin	CDH5	13.80	0.003	↑	10.51	0.0022	↑
Integrin, beta 3	ITGB3	8.86	0.021	↑	7.568	0.023	↑
Integrin, alpha V	ITGAV	0.186	0.009	↓	8.654	0.006	↑
Collagen, type IV, alpha 3	COL4A3	0.022	0.018	↓	0.0416	0.035	↓

***Growth factors and receptors***							

Vascular endothelial growth factor C	VEGFC	25.05	0.028	↑	19.84	0.034	↑
Hepatocyte growth factor	HGF	23.86	0.040	↑	8.476	0.0013	↑
Transforming growth factor, alpha	TGFA	16.34	0.035	↑	28.18	0.02	↑
Platelet-derived growth factor alpha polypeptide	PDGFA	11.88	0.02	↑	8.206	0.04	↑
TEK tyrosine kinase, endothelial	TEK	10.34	0.047	↑	0.248	0.007	↓
Ephrin-A3	EFNA3	9.65	0.037	↑	13.77	0.034	↑
Ephrin-B2	EFNB2	6.85	0.02	↑	9.254	0.043	↑
Neuropilin 2	NRP2	7.345	0.026	↑	0.045	0.013	↓
Tumor necrosis factor, alpha-induced protein 2	TNFAIP2	5.515	0.010	↑	16	0.003	↑
Jagged 1	JAG1	0.015	0.041	↓	0.0146	0.040	↓
Epidermal growth factor	EGF	0.211	0.012	↓	0.0963	0.006	↓

4 genes ITGAV, COL4A3, JAG1 and EGF were downregulated at 30 days for both short and long DLI PDT with exception of ITGAV in long DLI HY-PDT.

## Discussion

It is evident from the present study that short and long DLI PDT induces differential expression of angiogenic proteins and genes in bladder carcinoma tumors. In order to understand the *in vivo *uptake kinetics of HY, which depends on its physicochemical and pharmacokinetic properties, macroscopic and microscopic fluorescence of HY were examined. At 6 h post HY administration the intensity of both macro and microfluorescence in the tumor was significantly greater compared to 0.5 h. By 6 h post HY administration, the drug rapidly diffuses from the blood vessels and selectively accumulates in the tumor tissue and similar findings have been reported in radiation-induced fibrosarcoma (RIF-1) tumors [21]. Based on the distribution of HY in the tumor, PDT in our experiments was conducted at 0.5 h (short DLI) and 6 h (long DLI) to target the vasculature and tumor cells respectively. At short DLI, the damage is more vascular targeted and at long DLI greater cellular damage is expected. Our tumor regression experiments conducted in the bladder carcinoma xenograft model showed that short DLI PDT which causes greater vascular damage can improve tumor response compared to the tumors treated with long DLI PDT. Also the two time points 24 h and 30 days were chosen to study the immediate and long time effects of PDT on the angiogenic response.

To assess the status of vasculature, CD31 staining was used to visualize and enumerate the blood vessels. At 24 h short DLI PDT CD31 staining of congested nonfunctional blood vessels were observed showing evidence of maximum vascular damage and at long DLI PDT, functional blood vessels were noticed at 24 h post PDT demonstrating greater cellular destruction than vascular damage at longer DLI PDT. At 30 days the short DLI PDT effect on the tumor diminished and minimal staining was observed, however comparably higher CD31-stained blood vessels were noticed in long DLI PDT, suggesting neovessel formation and tumor regrowth. Similar to our studies, extensive vascular damage assessed by CD31 staining was observed by 24 h after PDT treatment in colon-26 murine tumors [22].

PDT-induced inflammatory changes are characterized by enhanced expression of a number of angiogenic molecules and proinflammatory cytokines as reported in earlier studies [17,19,20]. We investigated the expression of angiogenic proteins by employing western blotting and antibody arrays techniques. IL-6 protein was rapidly and strongly enhanced in the PDT treated tumors at 24 h suggesting a strong immune response which potentiates anti-tumor immunity. Our results confirm the enhancement of IL-6 post PDT as described earlier by Gollnick et al. [23]. Also upregulation of IL-6 was observed in Photofrin^® ^photosensitization of HeLa cells [24] and EMT6 cells [25]. Increased expression of VEGF, TNF-α, bFGF and IFN-α and -γ was observed in long DLI compared to short DLI PDT at 24 h post irradiation. VEGF expression could be upregulated by PDT induced hypoxia in the tumor cells as long DLI PDT causes greater cellular damage [19,26]. As TNF-α is secreted by resident macrophages and stromal cells following stimulation, the tumor cells under oxidative stress at 6 h DLI PDT could have enhanced the expression of TNF-α. Increased TNF-α after PDT has been reported by Gomer et al. [27], their group also demonstrated the upregulation of cytokines post photofrin PDT. Interestingly, we noticed suppression of TNF-α at 30 days post short DLI PDT, that may suggest minimal macrophage recruitment due to extensive vascular mediated damage, however this hypothesis warrants further research. On the other hand, it would be reasonable to hypothesize that longer DLI PDT that induces oxidative stress within the tumors cells could have caused the noted upregulation of angiogenic proteins such as bFGF, IFN-α and -γ. Our earlier study on nasopharyngeal carcinoma using HY-PDT showed upregulation of bFGF that has also been attributed to PDT induced hypoxia [28]. In the same way, upregulation of IFN-γ has been reported in a study whereby antigen presenting cells from PDT-treated mice exhibited an enhanced ability to stimulate T-cell proliferation and IFN-γ secretion [29].

The formation of new microvessels are mediated by the endothelial cell adhesion molecules such as integrins, selectins, cadherins and immunoglobulins [30] and the lack of appropriate cell contacts may even lead the endothelial cells to programmed cell death [31]. HY-PDT induces extensive vascular and cellular damage at 24 h time point, due to which an expected downregulation of cell adhesion molecules, cadherin 5, collagen alpha 1 and collagen alpha 3, intergrin alpha V and integrin beta 3 was observed. However, when the PDT induced damage diminished, upregulation of these molecules were noted. Studies conducted recently have demonstrated that reactive oxygen species produced during PDT eliminates highly proliferating cells by damaging membranes and affecting the extracellular matrix components profoundly, especially the collagen matrix [31,32].

Hepatocyte growth factor/scatter factor (HGF), a cytokine that stimulates tumor cell motility, invasion and angiogenesis [33], was overexpressed in all the four treatment groups. HGF receptor is a tyrosine kinase encoded by c-met and activation of HGF/c-Met pathway is shown to play a key role in tumor induced angiogenesis [34]. It has also been reported that patients with urinary bladder cancer have elevated levels of HGF in urine and bladder cancer tissue [35]. Thus, our data suggests a functional role of HGF in MGH bladder tumors. HGF also plays a prominent role in the induction of the proangiogenic factors PDGF, VEGF and IL-8 [36]. This suggests that the upregulation of PDGFA could possibly be a result of the transcriptional activation of PDGFA gene by HGF.

Significantly enhanced transcription of Ephrin A3 and Ephrin B2 was observed 30 days post short and long DLI PDT. These are classes of proteins that bind to the Eph receptors, a subset of the endothelial cell receptor tyrosine kinase (RTK), and promote cell attachment, cell-adhesion, migration, capillary tube formation and ultimately lead to tumor angiogenesis [37]. The Eph receptors also regulate integrin-dependent cell adhesion through activation of the c-Jun kinase via Nck-interacting Ste20 kinase in endothelial cells [38]. This further suggests that the downstream events in the Ephrin signaling can lead to enhanced angiogenesis even at 30 days post PDT.

Transforming growth factor-alpha (TGFA) is another growth factor that interacts with the same receptor as EGF and induces mitogenic and cell differentiating responses by binding to and activating surface EGF receptor (EGFR), while high transcription activity of TGF-α and EGFR has been reported in human tumors [39]. Though TGFA was downregulated immediately post short and long DLI PDT, EGF was found to be highly expressed at the initial time point. However, when TGFA is upregulated 30 days post PDT, EGF is downregulated. Thus it can be concluded that at any time point, the multiple EGFR downstream intracellular signaling cascades are activated either by TGFA or EGF in our HY-PDT treated tumors.

Neuropilin-2 (NRP-2) expression correlates with advanced tumor stage and grade [40]. Neuropilins are expressed in the tumor vasculature and in tumor cells, and its upregulation at 24 h post PDT can be correlated to tumor angiogenesis and tumor progression. Jagged 1 (JAG1) is a ligand for multiple Notch receptors and is involved in the mediation of Notch signaling that controls multiple cell differentiation processes [41]. Downregulation of JAG1, a notch ligand in all treatment groups could suggest that PDT could suppress the notch signaling cascade. TEK (tyrosine kinase, endothelial cell), also known as TIE-2, constitute a subfamily of receptor tyrosine kinase, known for its specific expression in vascular endothelial cells [42]. In the present study the TEK gene was found to be upregulated 30 days post short and long DLI PDT. Thus it can be speculated that the expression of this tyrosine kinase would further signal the downstream pathway leading to cell proliferation, migration and survival [43]. These results suggest that Ang1/Tie2 signaling plays an important role in endothelial cell survival post HY-PDT.

The enhanced angiogenic effect at 30 days post PDT can be attributed to the increased expression of angiogenic growth factors and receptors such as HGF, TGFA, PDGFA, VEGFC and TEK. The angiogenic process is often triggered by proangiogenic factors such as VEGF, PDGF, TGFA, EGF and bFGF [44]. VEGF is the most pivotal positive proangiogenic regulator detected in various tumors [45] and has the ability to elicit multiple responses depending upon the context of its expression and the presence of other growth factors. High VEGF mRNA transcription at 30 days post PDT could suggest its major role in tumor angiogenesis. Gomer et al. [17] have previously reported that PDT induced hypoxia triggers cytokine expression. Consistent with this study, we have reported that PDT induced inflammation and hypoxia upregulates various cytokines and proangiogenic factors, i.e., VEGF, bFGF and EGF [20].

## Conclusion

In summary, we have established that differential expression of genes could be a result of the interval between photosensitizer administration and light activation. Our results indicate that predominantly cellular targeting long DLI PDT can induce greater expression of angiogenic proteins compared to vascular targeting short DLI PDT. Collectively, we observed upregulation of 4 genes at 24 h post HY-PDT and 10 genes at 30 days post HY-PDT, indicating a greater need for combination therapy by including anti-angiogenic agents that target specific pathways.

## Methods

### Photosensitizer

The photosensitizer HY (Molecular Probes, OR, USA) was dissolved in dimethyl sulfoxide, DMSO (Sigma Aldrich Inc, St Louis Mo, USA) and diluted in phosphate-buffered saline (PBS) and administered intravenously at a dose of 5 mg/kg.

### Cell culture conditions and xenograft tumor model

Male balb/c nude mice, 6–8 weeks of age, weighing an average of 24–25 g were obtained from the Animal Resource Centre, Western Australia. To establish a murine xenograft model of human bladder carcinoma, the present study used MGH epithelial bladder cell line. Briefly, cells were cultured as a monolayer in RPMI-1640 medium. Approximately 3.0 × 10^6 ^MGH bladder cells suspended in 150 μl of Hanks' balanced salt solution (Gibco, USA) were injected subcutaneously into the lower flanks of the mice. The mice were kept for 10–14 days to allow the tumors to grow till they were palpably greater than 5–6 mm. The tumor volume was calculated using the following formula: volume = (π/6 × d1 × d2 × d3), where d1, d2 and d3 are tumor dimensions in 3 orthogonal directions. All procedures carried out in this study were approved by the Institutional Animal Care and Use Committee (IACUC), SingHealth, Singapore and were conducted in accordance with international standards.

### Fluorescence imaging

A dose of 5 mg/kg of hypericin was administered to mice through tail vein injections. The mice were anesthetized using 1:1 vol/vol cocktail of ketamine hydrochloride (Trittau, Germany) and valium (David Bull Laboratories, Australia). The skin overlaying the tumor was carefully removed to expose the tumor and imaging was performed at 0.5 and 6 h post hypericin administration. A fluorescence endoscope system (Karl Storz, Tuttlingen, Germany) was used to perform macroscopic fluorescence digital imaging. This system consists of a fluorescence detection unit, an illumination console, a video displaying and recording unit, and a computing system for image acquisition, display and processing. The D-Light system contains an excitation filter (375–440 nm) that allows high transmission in the violet part of the spectrum and blocks the rest of the visible part of the spectrum. The fluorescence can be detected since a large portion of the excitation light is being blocked by an observation filter (cut-off wavelength at 470 nm). An optical filter is located in the eyepiece of the fluorescence endoscope (Karl Storz, Hopkins II 0°, 4 mm) and it reduces blue light excitation that is diffusely back-scattered by the tissue and transmits red fluorescent light. The images thus obtained were analysed using the image analysis software, MicroImage 4.0. Images obtained in blue light needed to be further processed to obtain normalized fluorescence intensity images. The processing involves contrast enhancement of the original image. This is followed by hue extraction where the fluorescence color (red) is extracted. Following hue extraction, the thresholding and segmentation of the area of interest (AOI) was carried out. This gives us the red-black image, from the red-black image we obtained the relative intensity distribution over the tumor and the normal regions. Finally, the normalized intensity image, which is the ratio of the tumor fluorescence to the normal (blue) background, was obtained. The ratio of red intensity over blue intensity from 10 images per group was quantified using the software and the graph was plotted.

### Laser confocal fluorescence imaging

After intravenous administration of hypericin the animals were sacrificed at 0.5 and 6 h and tumors were subsequently extracted. The tumors were then snap frozen in liquid nitrogen. Cryosections of 20 μm thickness were obtained using a microtome cryostat (Cryo-Star HM 560 MV, Germany) and the sections were mounted onto slides. A laser confocal fluorescence microscope (Meta LSM 510, Carl Zeiss, Germany) was used to obtain confocal fluorescence images. A 488 nm Argon laser was used to excite the tissue. Fluorescence emissions in the wavelength range of 590–630 nm were spilt by a diachronic filter and detected through a band-pass filter (BP-610 nm, Omega Optical, USA). Each fluorescence image of any field was reconstructed from a sequence of 20 sequential optical images. Voltage gain, PMT voltage and sensitivity (contrast, brightness and filters) were fixed for all fields and slides imaged. Fluorescence data analysis was carried out using an image software package (Kontron KS400, version 3.0, Hallbergmoos, Germany). A total of 10 images, acquired from different tumor samples, were used to quantify fluorescence intensity. Briefly, digital quantification was carried out by using box superimposition of an area of 100 pixels, or contour super-imposition where the contour of the area of interest was outlined and the pixel intensities per unit area determined. This was done to determine the relative hypericin fluorescence between different regions of the same specimen.

### PDT treatment protocol

Animals were assigned to five different groups: (i) control (untreated tumor), (ii) short DLI PDT 24 h, (iii) long DLI PDT 24 h, (iv) short DLI PDT 30 days and (v) long DLI PDT 30 days, and each group comprised of 10 animals. Tumors from groups (ii) and (iii) were extracted at 24 h post PDT whereas tumors from groups (iv) and (v) were extracted at 30 days post PDT. The PDT treatment groups received intravenous injection of HY followed by irradiation with a light source consisting of broadband halogen light (Zeiss KL1500) fitted with a customized 560–640 nm band-pass filter. A light dosage of 120 J/cm^2 ^and 100 mW/cm^2 ^was used for PDT treatment. It should be noted that only the DLI was varied, the photosensitizer dose was maintained for all the treated groups.

### Tumor tissue lysate preparation

At the designated time points of 24 h and 30 days post PDT, the mice were humanely sacrificed. The tumor tissues were then harvested and immediately frozen in liquid nitrogen. The tissues were then crushed into powder in liquid nitrogen and ice-cold lysis buffer (T-PER, Pierce) with protease inhibitor (Complete Mini, Roche) was added and then kept on ice for 30 min. After subsequent centrifugation at 14,000 × *g *for 15 min at 4°C, supernatants (total tumor cell lysates) were stored at -70°C. The protein concentration was estimated by DC Bio-Rad assay in accordance with to the manufacturer's protocol.

### Immunohistochemistry

Tumor tissues from both control and HY-PDT treated animals were used for immunohistochemistry. Immunohistochemical staining was done using the Chemicon Blood vessel staining kit on formalin-fixed, paraffin-embedded control and HY-PDT tumor tissues. Sections were incubated with anti-CD31 (1:100) for 1 h followed by incubation with anti-rabbit secondary antibodies conjugated to horseradish peroxidase-linked labeled polymers. 3,3'-Diaminobenzidine-positive substrate-chromogen was added to sections and incubated for 5 minutes for color development. Finally, the slides were counterstained with Harris's haematoxylin for 5 seconds, dehydrated, and mounted. Primary antibody was replaced with normal mouse serum IgG_1 _(1:500) to confirm the specificity and served as negative control. Microvessels stained with CD31 were counted by light microscopy at X200 magnification. Any stained endothelial cell or cell cluster clearly separated from adjacent vessels was considered a single countable microvessel. Slides were examined by two independent observers. IHC scoring was performed based on the prevalence of CD31 staining within the tumor (No staining = 0, < 10% staining = 1, 10–25% = 2, 25–50% = 3, 50–75% = 4 and 75–100% = 5). Images from the slides were captured using image processing software (Kontron KS400, version 3.0, Hallbergmoos, Germany).

### Western blotting

For Western blot analysis, 30 μg of protein were resolved on 10–12% polyacrylamide-SDS gels and transferred onto a nitrocellulose membrane as described elsewhere. The membrane was immersed in blocking buffer (5% non-fat dry milk/1% Tween 20 in 20 mM TBS, pH 7.5) for 1.5 h at room temperature and incubated with the appropriate primary antibody i.e., mouse monoclonal anti-TNF-α (1: 50) and IFN-α (1:100) (Santa Cruz Biotechnology, Santa Cruz, CA, USA) and mouse monoclonal anti-human VEGF (1:100) (BD Pharmingen, BD Biosciences, CA, USA) solved in blocking buffer overnight at 4°C, followed by incubation with complementary secondary antibody. Proteins were detected by chemiluminescence (Supersignal, Pierce Technology, Rockford, IL, USA) and autoradiography using Hyperfilm ECL (Amersham Biosciences, UK).

### Detection of angiogenesis proteins using antibody array

Expression of angiogenesis proteins following HY-PDT were measured using the RayBio^® ^Human Angiogenesis Antibody Array 1.1 (RayBiotech Inc, USA) in control and PDT subjects (8 control and 8 HY-PDT) using one array per tissue sample. Only expressions of proteins relevant to this study are reported in this article. The components in the kit included protein array membranes, biotin-conjugated anti-cytokines, HRP-conjugated streptavidin and detection buffer. Briefly, the protein array membranes were immersed in blocking buffer, after which the membranes were incubated with tumor lysate for 2 h. After extensive washing the membrane was incubated with primary antibody and after further washing the HRP-conjugated streptavidin was added and incubated for another 2 h. After adding the detection buffer, the membranes were exposed to X-ray film (Hyperfilm ECL, Amersham Biosciences UK) and the signal was detected using film developer (Kodak M35 OMAT Processor). The intensities of the signal were quantified using a densitometer. By comparing the intensities of the signals, the relative expression levels of the angiogenic proteins were analyzed. The results were normalized to an internal positive control provided on each membrane. RayBio Analysis Tool, a program specifically designed for the analysis of antibody arrays, was used to plot the graphs.

### RNA isolation and cDNA preparation

Total RNA was extracted from tumor tissue using the commercially available Nucleospin RNA II kit (Macherey-Nagel, Germany). Briefly, the frozen tissue samples were crushed into powder using liquid nitrogen and lysis buffer, and β-mercaptoethanol was added to prepare the lysate. The lysate was then filtered and 70% ethanol was added to adjust RNA binding to the columns. Later DNA digestion was performed and pure RNA was eluted. RNA quality and purity was checked using UV Spectrophotometry and by detecting the ribosomal RNA integrity. Complementary DNA (cDNA) was synthesized from 2 μg DNA-free total RNA before PCR analysis.

### Gene expression profiling using real-time PCR array

The Human Angiogenesis RT^2 ^Profiler™ PCR Array (SuperArray, USA) was used to identify the expression of 84 key genes involved in modulating the biological processes of angiogenesis. The array includes growth factors and their receptors, chemokines and cytokines, matrix and adhesion molecules, proteases and their inhibitors as well as transcription factors, involved in angiogenesis. For each PCR evaluation, amplification and detection was performed with the ABI Prism 7000 Sequence Detection Systems (Applied BioSystems, USA). Average ΔC_t _value for each gene across triplicate arrays for each treatment group was calculated. ΔΔC_t _for each gene across control and experimental group were determined. Finally the fold change was computed for each gene from control and other groups as 2^(-ΔΔC_t_).

### Statistics analyses

Statistical analysis was performed using GraphPad Prism version 4.0 (GraphPad Software, Inc., San Diego, CA, USA). One-way ANOVA was used to analyze the variance and Bonferroni's multiple comparison test was used to compare the significance between all the groups for the various experiments. Dunnett's multiple comparison tests was used to analyze the difference between control and the other groups. A *p *value of < 0.05 was considered to be significant.

## Abbreviations list

PDT: photodynamic therapy; DLI: drug light interval; HY: hypericin; DMSO: dimethyl sulfoxide; PBS: Phosphate buffered saline; VEGF: vascular endothelial growth factor; TNF-α: tumor necrosis growth factor-alpha; IFN-α/γ: interferon-alpha/gamma; bFGF: basic fibroblast growth factor; IHC: immunohistochemistry; IL-6: interleukin-6; ECM: external cellular matrix; CDH5: cadherin 5 type 2, VE-cadherin; COL18A1: collagen type XVIII alpha 1; COL4A3: collagen; type IV alpha 3; ITGAV: integrin alpha V; ITGB3: integrin beta 3; HGF: hepatocyte growth factor; EFNA3: ephrin-A3; PDGFA: platelet-derived growth factor alpha polypeptide; EGF: epidermal growth factor; NRP2: neuropilin 2; EFNB2: ephrin-B2; JAG1: jagged 1; TNFAIP2: tumor necrosis factor alpha-induced protein 2; TEK: tyrosine kinase endothelial; TGFA: transforming growth factor alpha; VEGFC: vascular endothelial growth factor C

## Competing interests

The authors declare that they have no competing interests.

## Authors' contributions

RB designed, carried out the experiments, analysed data and drafted the manuscript, YYG, KCS supervised the project and commented on the manuscript

SSL performed western blotting and commented on the manuscript

WWC performed real-time PCR and corrected the final manuscript

MO obtained funding, supervised the project and corrected final manuscript

MAS commented on the manuscript

All authors read and approved the final manuscript.
